# Shone syndrome revealed by treatment-resistant hypertension

**DOI:** 10.1016/j.amsu.2021.102955

**Published:** 2021-10-16

**Authors:** Soumia Boulouiz, Amine Kossir, Fadoua Mouedder, Chaimae Miri, Nabila Ismaili, Noha El Ouafi

**Affiliations:** a Department of Cardiology, Mohammed VI University Hospital of Oujda, Mohammed First University of Oujda, Morocco; b Laboratory of Epidemiology, Clinical Research and Public Health, Faculty of Medicine and Pharmacy, Mohammed the First University of Oujda, Morocco

**Keywords:** High blood pressure, Coarctation of the aorta, Mitral parachute valve, Supramitral ring, Left superior vena cava, Case report

## Abstract

**Introduction:**

and importance: Shone complex is a congenital heart defect consisting of four obstructive defects in the left heart: a mitral supravalvular ring, sub-aortic stenosis, parachute mitral valve, and coarctation of the aorta (CoA), which affects only a small minority of people.

**Case presentation:**

We report the case of a 25-year-old woman with a past medical history of moderate mitral stenosis, since she was 10-year-old with uncontrolled high blood pressure, treated with nicardipine. admitted to our emergency department with high blood pressure: 190/80 mmhg, in whom The transthoracic echocardiography (TTE) revealed: sub-mitral membrane, with a single sub-papillary muscle, and coarctation of the aorta and the CT scan showed narrowed aortic arch and a left superior vena cava allowing to retain shone syndrome as the main diagnosis. The patient was treated with an antihypertensive treatment combining (perindopril/indapamide/amlodipine) while waiting for surgery.

**Clinical discussion:**

In this mini-review, we aim to describe this rare pathological condition its pathophysiological thoughts, and the way to diagnosis this complex early.

**Conclusion:**

Treatment required the coordinated efforts of a team of specialists. It could be either surgical with different method or by Trans catheter treatments.

## Highlights

• The shone complex is a very rare congenital heart disease.
• The long-term prognosis with this complex is poor, with a mortality rate of 24%–27%.
• Minor mitral valve pathology patients should undergo a thorough diagnostic examination since they may have other obstructive lesions of the left outflow tract.
• Individual lesions differ in clinical presentation and severity, making correct diagnosis complex.
• Surgical interventions and outcomes in adult patients have not been reviewed as well as in children.

## 1 Introduction

The shone complex is a very rare congenital heart disease, with only a few adult cases [1,2]. It was reported for the first time by Shone et al. as a complete form with four obstructive involvements in the left heart: supramitral ring, a sub-aortic stenosis, a parachute mitral valve (PMV), and coarctation of the aorta (CoA) [3]. It includes vast anatomical and physiological varieties, from mild lesions that do not need treatment to severe forms that would often require surgery. The Long-term prognosis with this syndrome is poor, with a mortality rate of 24%–27% [4]. We report the case of a 25-year-old female, who suffered for over 4 years from a resistant-treatment hypertension, and a mitral stenosis. Then, she was diagnosed as shone syndrome. Through this case-report, we aim to explain, the pathophysiology of this syndrome, diagnostic approaches, its prognosis, and the different therapeutic strategies suggested.

Our case has been reported in line with THE SCARE 2020 criteria [5].

## 2 Patient information

**Fig. 1:**
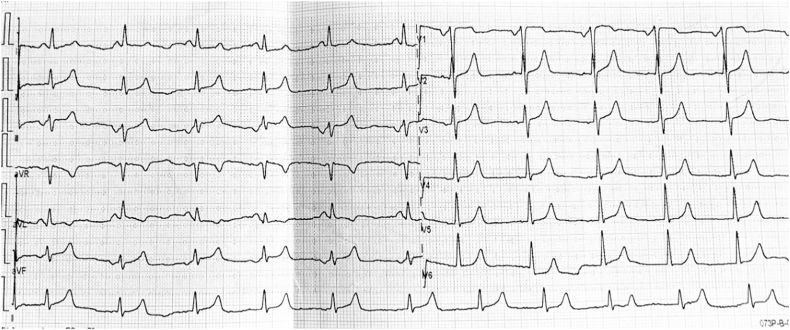
ECG showed a coronary sinus rhythm with a Heart Rate at 75 beats per minute, with left atrial hypertrophy.

A 25-year-old female patient with a past medical history of mitral stenosis for fifteen years and a treatment-resistant hypertension with nicardipine, without family history or psychosocial history. During her pregnancy, she presented severe high blood pressure without proteinuria then she was referred to our department from a gynecologist. The clinical examination revealed a blood pressure at 190/95 mmHg in upper limb and 100/60 mmHg in lower limb, left parasternal holosystolic murmur, with posterior irradiation, weak pulse in both lower limbs. The ECG showed a coronary sinus rhythm with a Heart Rate of 75 beats per minute, with left atrial hypertrophy (Fig. 1).

**Fig. 2:**
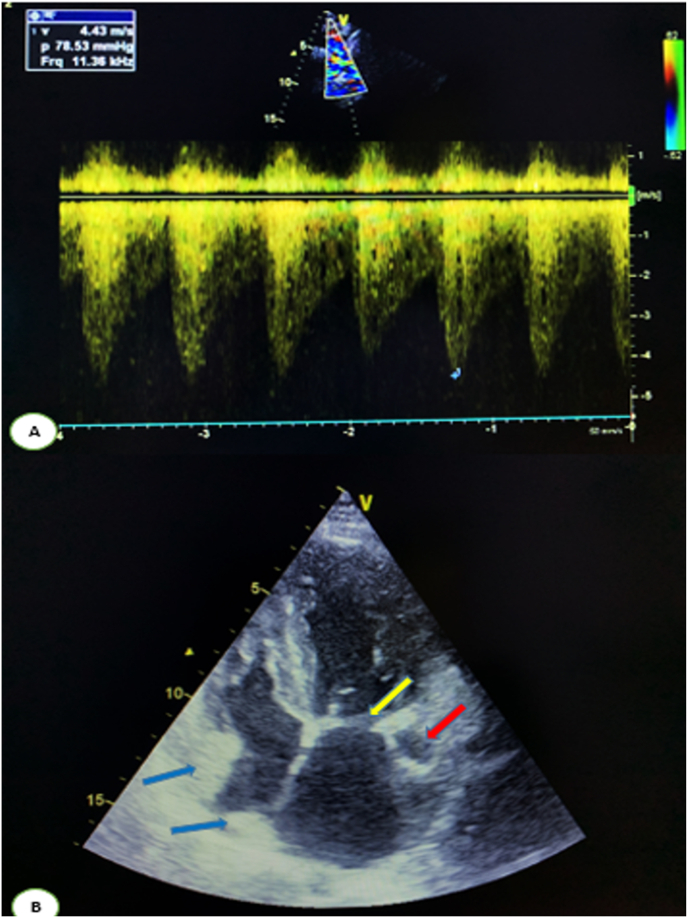
**(A)** Echocardiographic suprasternal view showed the presence of coarctation of the aorta with V max at 4.43 m/s with a mean gradient at 79 mmHg and an extended speed reduction during diastole**. (B)** Apical 4-chamber view, demonstrating sub-mitral membrane (yellow arrow), with a single sub-papillary muscle, two non-motile rounded hyper-echogenic formations in the right atrium (blue arrow), suggest embryonic residue, with a dilated coronary sinus (red arrow). (For interpretation of the references to colour in this figure legend, the reader is referred to the Web version of this article.)

The transthoracic echocardiography (TTE); showed an undilated left ventricle, hypertrophied (posterior wall at 13 mm), of good overall systolic and segmental function left ventricle fraction at 57%, sub-mitral membrane, with a single sub-papillary muscle, an attachment defect of the mitral valves responsible for a minimal leakage and a mitral stenosis under the tight valve (the mean gradient at 12 mmHg). Contrasting with the mitral surface, which was at 2 cm ^2^ by planimetry 2.2 cm^2^ by pressure half time (PHT). Left-right shunt between the aorta and the right ventricular, outflow responsible for a maximum gradient at 164 mmHg and Vmax at 6.42 m/s suggesting a small perimembranous ventricular septal defect or aorto right ventricular fistula. Tricuspid aortic valve; left atrium dilated free of echo, Dilated coronary sinus, two non-motile rounded hyper-echogenic formations in the right atrium, measuring 21/16 mm. The second 10/16 mm suggest embryonic residue with a dilated coronary sinus. (Fig. 2B). Undilated right ventricle with, good systolic function, No sign of pulmonary hypertension. The suprasternal view showed the presence of coarctation of the aorta with V max at 4.43 m/s with a mean gradient at 79 mmHg and an extended speed reduction during diastole (Fig. 2A), without a patent ductus arteriosus.

**Fig. 3:**
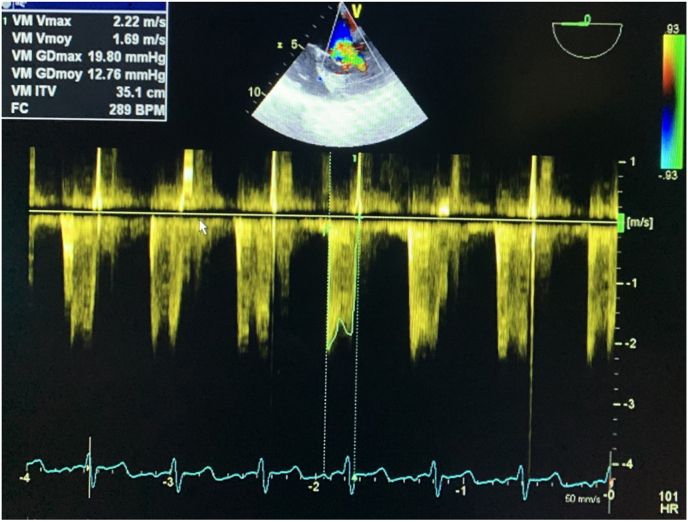
Trans esophageal echocardiography showed the same anomalies on The TTE a sub-mitral membrane, with a single sub-papillary muscle, an attachment defect of the mitral valves responsible of a mitral stenosis under the tight valve (the mean gradient at 12 mmHg) while the mitral surface was 2 cm ^2^ by planimetry on the TTE.

*Trans*-esophageal echocardiography (TOE) showed presence of a sub-mitral membrane, and a single papillary muscle, with attachment defect of the mitral valves responsible for a tight sub-valve mitral stenosis (mitral surface at 2 cm^2^ by planimetry and 2.2 cm^2^ by PHT, Gmoy at 12 mmHg) and minimal leakage. It showed also the presence of a left-right shunt between the aorta and the right ventricular flushing chamber responsible for a max Gradient at 164 mmHg and Vmax at 6.42 m/s: aorto RV suggested a small perimembranous ventricular septal defect (Fig. 3).

**Fig. 4:**
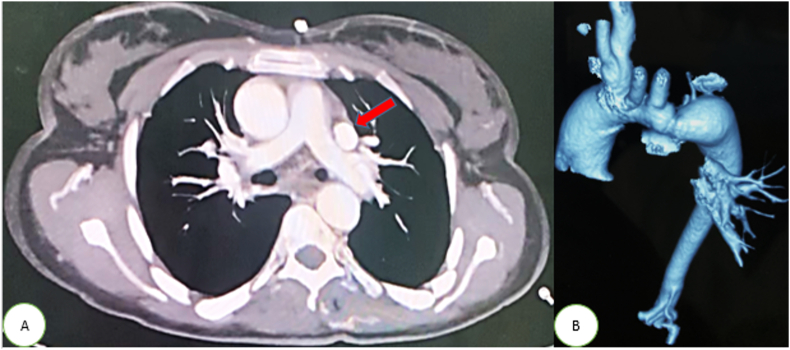
(A) Computed Tomography Chest showing persistent left superior vena cava (Red arrow) (B) 3D reconstruction of Computed tomography image showing an aspect of the narrowed aortic arch with a diameter of 6 mm, measured 9 mm bellow the origin of the left subclavian artery. (For interpretation of the references to colour in this figure legend, the reader is referred to the Web version of this article.)

A complementary thoracic CT angiography showed an aspect of narrowed aortic arch with a diameter of 6 mm, measured 9 mm bellow the origin of the left subclavian artery (Fig. 4B), and the left superior vena cava (LSVC) was also found (Fig. 4A).

The patient is actually on anti-hypertensive therapy (perindopril/indapamide/amlodipine) with persistence of borderline blood pressure and referred to cardiovascular surgery.

## 3 Discussion

Shone syndrome, first identified by John Shone in 1963 [3] as a series of barrier levels at the entrance and exit of the left ventricle, is an infrequent entity with a frequency of 0.7% of congenital heart disease. Its complete form associates a supra-mitral valve ring, sub aortic stenosis, parachute mitral valve (PMV), and coarctation of the aorta. Otherwise, the incomplete form, which is the most common, consists of a left ventricle inlet defect (PMV, Mitral stenosis congenitally or supravalvular mitral ring) associated with at least one left ventricular escape lesion (bicuspid aortic valve, subvalvular aortic stenosis, coarctation of the aorta) [6].

Coarctation of the aorta associated with mitral valve anomalies is described in 20–59% of cases, while the supravalvular annulus of the mitral valve and other defects in almost 90% [7]. Therefore, finding these defects should prompt searching for other cardiac abnormalities. Few interatrial or interventricular communication cases have been described in association with this complex [8,9]. Their presence may have contributed to a delay in the presentation and to the worsening of symptoms [10]. The pathogenesis of coarctation in this complex could be explained by a hemodynamic theory where perimembranous ventricular septal defect and patent ductus arteriosus or sub-aortic or abnormal vessel stenosis (left superior vena cava) leads to reduced aortic flow during fetal life and therefore hypoplasia of the isthmic region.

The presence of left superior vena cava (LSVC) in association with LV obstacles was reported in 39.6% of cases, as Agniletti et al. [8], found.this correlation might be explained by the mechanical impact of this last on the growth of the left heart, increasing the occurrence of obstructive involvements on the left side [7]. This anatomical variant must be identified during open-heart cardiac surgery, to use appropriate cannulation procedures, to redirect the vast volume of systemic venous blood that reaches the heart via the coronary sinus [10]. In patients with Shone complex, the presence of an LSVC should be ruled out before a pacemaker is implanted. According to a report by Sajid et al. 18% of patients had an arrhythmia or bradycardia that necessitated the implantation of a pacemaker or defibrillator [11].

Goswami et al. mentioned in a case of a young pregnant woman at 25 weeks gestation that the shone syndrome mimicked a preeclampsia [12]. This is consistent with our experience, in which arterial hypertension worsened during pregnancy despite consistently negative proteinuria.

Individual lesions differ in clinical presentation and severity, making correct diagnosis complex. Surgical interventions and outcomes in adult patients have not been reviewed as well as in children [6].

As she was 10 years old, our patient was only diagnosed with mitral stenosis, CoA should be detected earlier, and the restoration could be necessary if she underwent a rigorous examination. Therefore the cardiologist must have the reflex to look for an aortic anomaly, each time he finds a mitral anomaly, also it is necessary to look for left ventricular obstacles when he finds a dilated coronary sinus. Any discordance between the mitral surface and the transmitral gradient sugget a subvavular membrane and it must be researched, in our case the mean gradient was at 12 mmHg while the mitral surface was at 2.2 cm.^2^

Our patient was lucky that she had not complications with long-standing arch hypoplasia, aortic stenosis, or regurgitation. Therefore, it is vital to search for other accompanying left-sided probable heart impairments in patients with parachute mitral valve deformity.

According to the literature, most children with Shone complex had their coarctation repaired firstly, then their valve replaced or repaired. In a few cases, some intracardiac impairments were repaired at the same time [2,13]. Surgery should be performed as soon as possible before any signs of pulmonary arterial hypertension (PAH) occur, without forgetting; that the structure of the PMV and the supravalvular membrane must be precisely determined before the Surgery [5] Repairing, the mitral valve rather than replacing it is a safer surgical option because it removes the need for lifetime anticoagulation. Replacement should only be used in cases where the repair has failed [14].

Due to imbalanced chordal attachment and supravalvar blockage, the most common forms of mitral obstructive lesions in this condition (parachute deformity and supramitral ring) are not susceptible to balloon angioplasty, leaving surgery as the only reasonable alternative [15,16]. Although recurrence has been reported in one instance, resection of the Supramitral fibrous rings is simple [17].A reconstructive technique including of dissecting the papillary muscle and releasing and separating chordae tendinae to open the destroyed chordal gaps and enlarge the effective mitral orifice resulted in good hemodynamic outcomes [18].

The most recent recommendations for the management of CoA, as issued by the european society of cardiology and the american heart association/american college of cardiology [19], interventional therapy is indicated in all patients with a non-invasive blood pressure difference >20 mmHg between upper and lower limbs, regardless of symptoms but with upper limb hypertension (>140/90 mmHg in adults), pathological blood pressure response during exercise, or signiﬁcant LV hypertrophy (class I, level C). Independent of the PPG, hypertensive patients with ≥50% aortic narrowing relative to the aortic diameter at the level of the diaphragm should also be considered for treatment (class IIa, level C).

The treatment of CoA could be either surgical with different method like resection with end-to-end anastomosis, patch aortoplasty, subclavian flap aortoplasty or transcatheter treatments can be used to treat native CoA as well as re-CoA or aneurysm development following initial repair. Nowadays Stenting using a balloon angioplasty with concomitant stenting is now regarded the best therapeutic approach for adolescent and adult patients with native or re-CoA [20].

The severity of mitral valve obstruction still the main predictor of prognosis [1,2], the long-term prognosis with this complex is poor, with a mortality rate of 24%–27% [4]. In patients with Shone complex, good outcomes after surgical repairing were marked, especially if it is performed early enough before PAH develops [12].

## 4 Conclusion

This case demonstrates that the Shone complex is an unusual and under-diagnosed diagnostic entity. Even minor mitral valve pathology patients should undergo a thorough diagnostic examination since they may have other obstructive lesions of the left outflow tract. Previous studies have described that children who had coarctoplasty or repair in early childhood or as part of a staged procedure, but our situation is unusual. It represents an incomplete type of Shone complex that was discovered in adulthood.

## Provenance and peer review

Not commissioned, externally peer-reviewed.

## Sources of funding

This research did not receive any specific grant from funding agencies in the public, commercial, or not-for-profit sectors.

## Ethical approval

The ethical committee approval was not required give the article type (case report).However, the written consent to publish the clinical data of the patients was given and is available to check by the handling editor if needed.

## Consent

Written informed Consent was obtained from the patient for publication of this case report and accompanying images. A copy of the written consent is available for review by the Editor-in-Chief of this journal on request.

## Author contributions

SOUMIA BOULOUIZ: study concept or design, data collection, data analysis or interpretation, writing the paper.

AMINE KOSSIR: data analysis or interpretation, writing the paper.

FADOUA MOUEDDER: : data analysis or interpretation.

CHAIMAE MIRI: data analysis or interpretation.

NABILA ISAMAILI: supervision and data validation.

NOHA EL OUAFI: supervision and data validation.

## Registration of research studies

This is not an original research project involving human participants in an interventional or an observational study but a case report. This registration is was not required.

## Guarantor

SOUMIA BOULOUIZ.

## Declaration of competing interest

The authors state that they have no conflicts of interest for this report.
